# 4,8-Dicarboxyl-8,9-iridoid-1-glycoside inhibits apoptosis in human osteoarthritis chondrocytes via enhanced c-MYC-mediated cholesterol metabolism in vitro

**DOI:** 10.1186/s13075-023-03217-1

**Published:** 2023-12-11

**Authors:** WeiBing Wang, HaiMin Mai, Huang Xu, BaoSheng Jing, CuiYu Yu, XiaoTing Li, DanGui Chen, Yuan Huang, MeiMang Shao, Tao Pan

**Affiliations:** 1 Department of Anesthesiology, Anqing Municipal Hospitals, Anhui Medical University, Anqing, 246000 People’s Republic of China; 2 Department of Orthopedic, The Sixth Affiliated Hospital, Sun Yat-sen University, Guangzhou, 510008 People’s Republic of China; 3 Department of Orthopedics, Anqing Municipal Hospitals, Anhui Medical University, Anqing, 246000 People’s Republic of China; 4 Department of Hematology, Anqing Municipal Hospitals, Anhui Medical University, Anqing, 246000 People’s Republic of China; 5 Department of Science and Education, Anqing Municipal Hospitals, Anhui Medical University, Anqing, 246000 People’s Republic of China; 6 Department of Orthopedic, Anqing Municipal Hospitals, Anhui Medical University, Anqing, 246000 People’s Republic of China

**Keywords:** Osteoarthritis, Borojoa, Cholesterol metabolism, Apoptosis, Autophagy

## Abstract

**Background:**

Osteoarthritis (OA) is a degenerative disease related to cholesterol metabolism disorders. However, current therapies for OA are insufficient and no convincing disease-modifying OA drugs exist. Therefore, we aimed to elucidate the mechanism by which borojoa iridoid glycoside (BIG) inhibits chondrocyte apoptosis in OA.

**Methods:**

Borojoa pulp was heated to 70 °C, and the main active substance in borojoa, BIG, was extracted by fractionation at an ultraviolet 254-nm absorption peak. Chondrocytes were identified by immunohistochemistry and visualized by immunofluorescence confocal microscopy. The proliferation of chondrocytes cultured with BIG was determined by MTS assay. The apoptosis of chondrocytes cultured with BIG was tested by Annexin V-FITC/PI, and the cytokine, protein, and cholesterol levels in chondrocytes were detected by ELISA, RT‒qPCR, Western blot, and biochemistry analyses. Protein‒protein interactions were verified by a coimmunoprecipitation (Co-IP) assay.

**Results:**

BIG promoted chondrocyte proliferation and reduced apoptosis in vitro. BIG induced an alteration of the total RNA profiles in chondrocytes, and bioinformatic analysis showed that BIG inhibited chondrocyte apoptosis by promoting c-MYC expression; KEGG analysis confirmed that BIG-inhibited apoptosis was enriched in the cell cycle pathway. Flow cell cycle experiments confirmed that BIG promoted chondrocyte proliferation by significantly increasing the S phase cell number. The c-MYC inhibitor 10058-F4 stimulated the increased expression of IL-1β, IL-6, TNF-α, and AGEs and suppressed the cholesterol metabolism, which promoted chondrocyte apoptosis and autophagy. Co-IP analysis showed that BIG promoted the interaction of c-MYC and CH25H, Bcl-2, which suggests that BIG could inhibit chondrocyte apoptosis in part by enhancing c-MYC-mediated cholesterol metabolism.

**Conclusions:**

This study confirmed that BIG promotes chondrocyte proliferation and inhibits apoptosis and autophagy, and BIG improving OA is associated with cholesterol metabolism. The results identify a potential mechanism by which BIG enhances c-MYC-mediated CH25H regulation of cholesterol metabolism in vitro and suggest that BIG might be a promising new drug against OA.

**Supplementary Information:**

The online version contains supplementary material available at 10.1186/s13075-023-03217-1.

## Introduction

Osteoarthritis (OA) is the most common degenerative disease and affects 7% of the global population, more than 500 million people worldwide, including a disproportionate number of women; OA was the 15th highest cause of years lived with disability (YLD) worldwide and was responsible for 2% of YLD in 2019 [1]. These data may underestimate the true size of the problem, and the progress achieved in OA treatment is very limited. The pathophysiological concept of OA is still evolving, from being seen as a cartilage limitation to a multifactorial disease affecting the whole joint, the complex relationship between local and systemic factors regulates its progression, leading to a common ultimate pathway of joint destruction [2]. Joint replacement is the standard treatment for end-stage OA; however, patients who have not reached the point of surgery suffer from joint stiffness and pain. None of the available pharmacologic therapies is effective in preventing or delaying OA progression [3], and the main treatment aims are to reduce pain and symptoms and improve joint functional capacity [4, 5]. Thus, there is an urgent need to explore disease-modifying OA drugs (DMOADs) that can alleviate the development of OA [6]. The unsatisfactory therapeutic effects of painkillers are related to drug dependence and overdose, and lifestyle modification seems to offer the most promising avenue to prevent OA [7]. The Lancet Commission on OA will provide a blueprint for a world free from OA-related disability [8].

The underlying mechanism of OA remains unclear. Recent studies have indicated that OA is related to cholesterol metabolism [9–11]. Metabolic abnormalities and obesity promote the progression of OA [12, 13]. In particular, abnormal lipid metabolism is related to OA, and OA has recently been considered a metabolic syndrome disease [14]. Cholesterol metabolism can affect chondrocyte growth and proliferation and regulate chondrocyte apoptosis. Studies have shown that statins are potential disease-modifying treatments for OA, supporting a direct role for cholesterol homeostasis in OA pathogenesis [15, 16].

Borojo (*Borojoa patinoi* Cuatrec) is a plant species belonging to the Rubiaceae family that is endemic to the rainforests of the Pacific region of Colombia. The fruit is used in traditional medicines with supposed antihypertensive, antitumoral, diuretic, immunological, anti-inflammatory, healing, and aphrodisiac effects [17]. Previous studies have shown that borojoa has antioxidant, antibacterial, and antitumour activities in vitro [18, 19]. Antioxidative capacity is considered to be closely related to lipid metabolism and can improve lipid metabolism-related diseases such as Alzheimer’s disease, cardiovascular disease, kidney disease, and diabetes [20, 21]*.* However, there are few reports on how borojoa and its extracts with antioxidant capacity may play a role in cholesterol metabolism.

During the analysis of borojoa fruit powder, we detected a small molecule compound, BIG, with antioxidant capacity that may be involved in cholesterol metabolism and inhibition of chondrocyte apoptosis. The aim of the present work was to determine (i) whether the small-molecule compound BIG inhibits chondrocyte apoptosis in OA and (ii) whether BIG inhibits apoptosis in OA by promoting cholesterol metabolism.

## Materials and methods

### Preparation and characterization of BIG from the borojoa fruit

Referring to our previous methods for extraction and isolation of BIG [22], in brief, borojoa fruit pulp was heated to 70 °C, and the main active substance of borojoa, BIG, was extracted by fractionation at an ultraviolet 254-nm absorption peak. The structures of the active extracts were analysed and characterized by nuclear magnetic resonance and infrared spectroscopy. The chemical name of BIG is 4,8-dicarboxyl-8,9-iridoid-1-glycoside, and the molecular formula is C_16_H_20_O_11_. The precise molecular weight is 388.10. The chemical structure is shown in Fig. 7O.

### Cell culture

#### Tissue samples collection

OA chondrocytes were isolated from the cartilage collected from osteoarthritis patients undergoing total knee replacement surgery. Briefly, the cartilage was cut into pieces and washed with PBS containing penicillin‒streptomycin. The cartilage was then incubated with pancreatin for 20 min and collagen, type II, alpha-1 (COL2A-1) for 2 h under slight vibration. The cells were collected and cultured with low-glucose DMEM in an incubator with 5% CO_2_. The medium was changed three times a week. The procedures were approved by the Ethics Committee of AnQing Municipal Hospital, Anhui Medical University, and all of the patients gave written informed consent.

#### Cell apoptosis analysis

Chondrocytes were cultured with or without BIG for 1, 5, and 9 days before conducting cell flow cytometry analysis. Cell apoptosis was detected by flow cytometry using an Annexin V-fluorescein isothiocyanate/propidium iodide (FITC/PI) apoptosis assay kit (Keygen, China). Briefly, cells were cultured with or without 100 μM BIG for 1, 5, or 9 days. The cells were harvested and washed with cold PBS and then stained with cold staining buffer. Cells for flow cytometric analysis were washed with PBS and centrifuged at 1000 rpm for 5 min. After resuspension in binding buffer, the solution was stained with fluorescein iso-thiocyanate-labelled Annexin V and PI for 15 min and then analysed using a FACS flow cytometer (BD Calibur, USA).

### Cell proliferation and antioxidant analysis

#### Cell proliferation analysis

Cells were cultured with BIG in 96-well plates for 1, 3, 5, 7, and 9 days, and cell proliferation was investigated by Cell Counting Kit-8 (CCK8) assay (Dojindo, Japan) according to the manufacturer’s instructions. The media was removed, and the cells were washed with PBS twice before 100 μL fresh medium containing 10 μL CCK8 was added. The plates were incubated for 3 h at 37 °C, and the optical density (OD) was measured using a microplate reader (Multiscan MK3, Thermo Fisher Scientific).

#### RT‒qPCR analysis

Total RNA was extracted from cells using TRIzol Reagent (Invitrogen, USA). cDNA was synthesized using a PrimeScript II 1st Strand cDNA Synthesis Kit (TaKaRa) following the manufacturer’s protocol. Real-time quantitative PCR(RT‒qPCR) was conducted using SYBR® Premix Ex Taq™ II (TaKaRa). The relative expression level of each gene was determined using the 2^−△△Ct^ method and normalized to β-actin in the same sample. The primers used in qPCR are listed in Table 1.

**Table 1:** RT‒qPCR primer sequences

Target gene	Primer sequence
CH25H	F: GTGGTCAACATCTGGCTTTC
	R: GCGAAGTTGCAGTTAAAGTG
CYP7B1	F: TATGTGCACGAGGACCTTGA
	R: TGACCTGTTGACTGCAGCAA
RORα	F:AGCACATAAACTGGGATGGG
	R:CAGTTCTTCTGACGAGGACG
MMP13	F: AGGAGCATGGCGACTTCTAC
	R: AGGGTCCTTGGAGTGGTCAA
MMP3	F: GCCAACTGTGATCCTGCTTT
	R: CCACGCCTGAAGGAAGAGAT
COL2	F: TTGGACGCCATGAAGGTTTT
	R:AGCTGAAATGGAAGCCACCT
Aggrecan	F:TGGGTCTGGAGTAGAAGTATA
	R: GTTAGCTTCGTGGAATGCA
18s	F: CCTGGATACCGCAGCTAGGA
	R:GCGGCGCAATACGAATGCCC

18s rRNA internal reference

*F* forward, *R* reverse

### Western blotting and coimmunoprecipitation (Co-IP)

Cells were harvested in RIPA buffer with protease inhibitor and phosphatase inhibitor. The total protein concentration was determined using a BCA protein assay kit (Pierce, Thermo Scientific, USA). Equal quantities of proteins were electrophoresed by SDS‒PAGE, and the separated proteins were transferred to a PVDF membrane (Millipore, Corporation, MA). The membrane was blocked with 5% defatted milk for 1 h at room temperature before incubation with the primary antibody overnight at 4 °C. Anti-BAX, anti-B-cell CLL/lymphoma 2 (Bcl-2), anti-cleaved-caspase3, anti-caspase3, and anti-GAPDH were purchased from Abcam. Binding was detected using HRP-conjugated secondary antibodies (1:2000; Bio-Rad Laboratories, Hercules, CA), and labelled proteins were visualized using an ECL chemiluminescence reagent. As a control, the expression of GAPDH (Santa Cruz Biotechnology, Santa Cruz, CA) was evaluated. The band intensities were analysed with the ImageJ software. For Co-IP, the procedures were performed according to the manufacturer’s instructions (26419, Thermo Fisher, USA).

### Immunofluorescence staining

The cells were fixed with 4% paraformaldehyde for 20 min, washed twice with PBS containing Triton X-100 for 5 min, and then blocked with 1% bovine serum albumin for 30 min. Next, the cells were incubated with Bcl-2 and cholesterol-25-hydroxylase (CH25H) antibodies (1:200 dilutions, Abcam) at 4 °C overnight. After three washes with PBS, the cells were incubated with a secondary antibody conjugated with fluorescence for 90 min at room temperature (Beyotime, China). The nuclei were counterstained with DAPI (Beyotime, China). Images were obtained under a BX51 microscope (Olympus, Tokyo, Japan).

### ELISA analysis

Inflammation factors were measured by ELISA. The concentration of total protein was determined using a BCA protein assay kit. The levels of tumour necrosis factor (TNF)-α, interleukin (IL)-1β, and interleukin (IL)-6 in the cell culture supernatants were quantified using an ELISA kit according to the manufacturer’s protocol (Cusabio, China).

### TC and FC analysis

At 1, 5, and 9 days of cell culture with or without BIG, the media were collected for analysis of the total cholesterol (TC) and free cholesterol (FC) content using TC and FC analysis kits (Suobio, China), respectively.

### Statistical analysis

Data are presented as the mean ± SEM and were statistically evaluated by one-way ANOVA between the control group and multiple groups, followed by the Kruskal‒Wallis test. Statistical analyses were conducted using the IBM SPSS 27.0 software. Significance was measured at the following thresholds: *P < 0.05, **P < 0.01, ***P < 0.001, and ns refers to not significant.

## Results

### Identification of chondrocytes

**Fig. 1:**
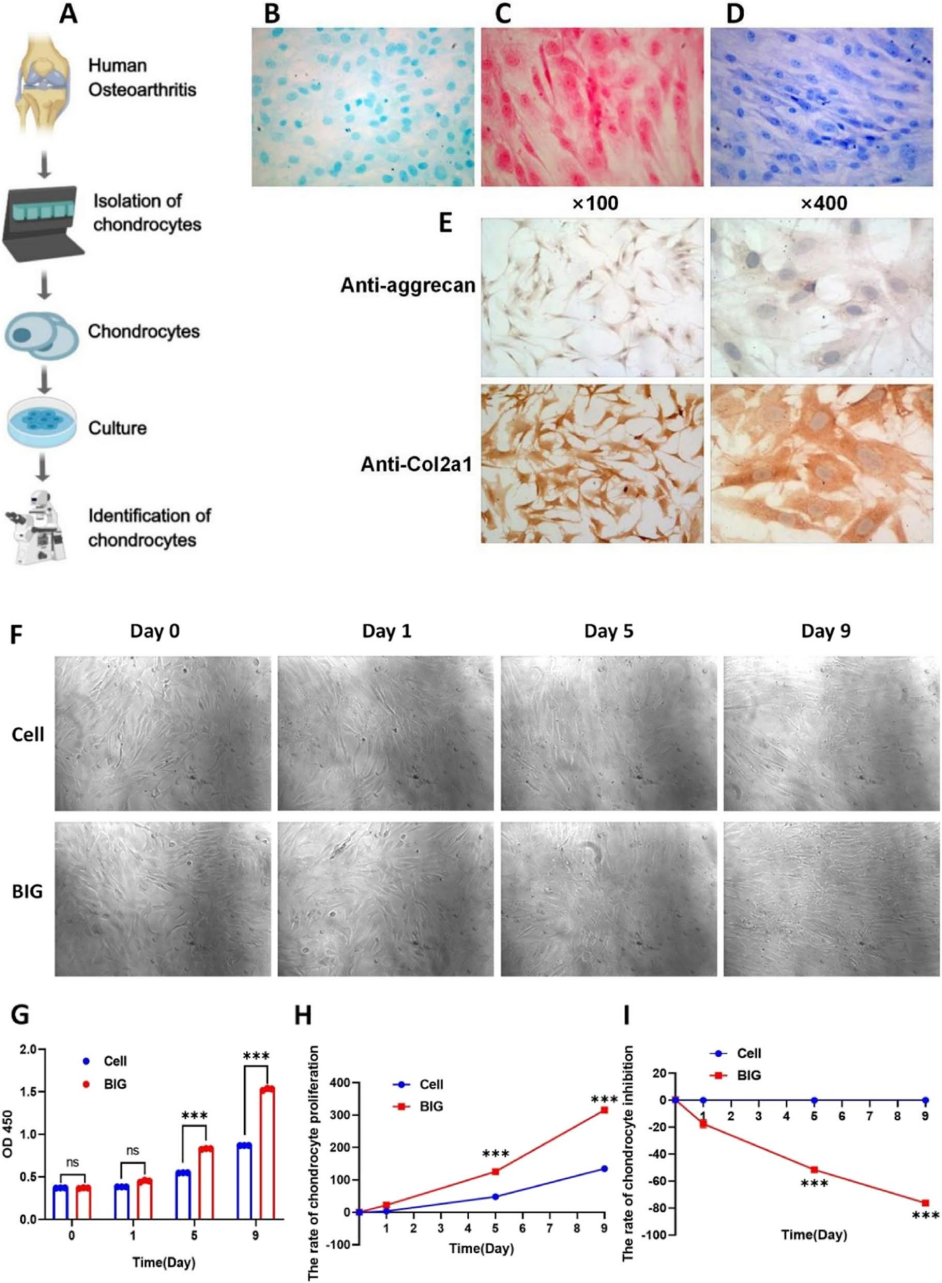
BIG can effectively promote the proliferation of OA chondrocytes in a duration-dependent manner. **A** Cells from the tibial plateau tissue were isolated and cultured. **B** The cytoplasm and cell membrane of chondrocytes were dark blue, proving that the chondrocytes secreted and synthesized proteoglycans. **C** The morphology of the red-stained cells was spindle and polygonal, and the nucleus was partial to one side and close to the cell membrane. The fibres of the cells were longer, and the cells were red without green, which shows that the cultured cells secreted proteoglycans. **D** Toluthanidine blue staining showed purplish-red allochromatic granules with a small number of granules around the cells. Granules were apparent (× 400). **E** During anti-aggrecan staining, purple abnormal staining granules were found in the chondrocytes, and a small amount of abnormal staining granules appeared around the cells; this phenomenon was more obvious in the formed cell nodule area. After anti-Col2a1 IHC staining, the chondrocyte cytoplasm was stained brown-yellow. The nuclei were uncoloured, and brownish-yellow particles were also observed in the extracellular matrix. **F** Microscopic pictures showed that BIG promotes chondrocyte proliferation and secretion matrix in chondrocytes (× 400). **G** The OD 450 value confirmed that BIG increased the concentration of chondrocytes. **H** The CCK-8 test confirmed that BIG effectively promoted the proliferation of chondrocytes. I BIG produces cytotoxicity in a time-dependent manner. Data are presented as the mean ± SEM (*n* = 3) and were analysed by the Kruskal‒Wallis test, **P < 0.01, ***P < 0.001

Abandoned tibial plateau bone tissue from OA patients undergoing total knee replacement was collected. After tissue separation was performed, cell culture, Alcian blue staining, redness staining, and toluidine blue staining confirmed the secretion and synthesis of proteoglycans by the chondrocytes. Immunohistochemical staining confirmed the characteristic manifestations of proteoglycans and Col2a1 in the chondrocytes (Fig. 1E).

### BIG promotes chondrocyte proliferation and cytotoxicity assay

Our previous study confirmed that the concentration of BIG ranging from 0 to 500 µg/ml had no effect on cell activity [22]; moreover, BIG inhibited chondrocyte apoptosis and autophagy in OA in a concentration-dependent manner ranging from 0 to 400 µg/ml, so we chose a concentration of BIG of 400 µg/ml. Coculture of chondrocytes with 400 μg/ml BIG resulted in a gradual increase in the chondrocyte concentration in the BIG group (Fig. 1G), the CCK-8 assay showed a temporal dependency of BIG in promoting chondrocyte proliferation (Fig. 1H), and BIG produces cytotoxicity in a time-dependent manner (Fig. 1I). Cell morphological observation confirmed that BIG has a strong ability to promote the proliferation of chondrocytes, secretion of matrix, and rapid cell proliferation. The cell group showed scattered spindle and polygonal cells around the degenerative cartilage tissue block, fusion phenomenon, and isolated sheets. On day 9, the cell volume began to increase, some finger processes appeared at the edge, the nucleus became larger, the cell membrane and cytoplasm were not clear, the proliferation rate slowed down, and the cells were fibrocell-like. In the BIG group, most cells began to appear, increased in size, and elongated to form protrusions after 24 h. The early cells were generally polygonal and had a round or oval nucleus. On day 5, the clustering of cells maintained round or oval growth, and overlapping growth phenomena appeared. On day 9, cell proliferation gradually resulted in fusion to form a monolayer, and the cells grew well and had a strong ability to proliferate, differentiate, and secrete matrix (Fig. 1F).

### BIG inhibits apoptosis of chondrocytes in OA by promoting cholesterol metabolism

**Fig. 2:**
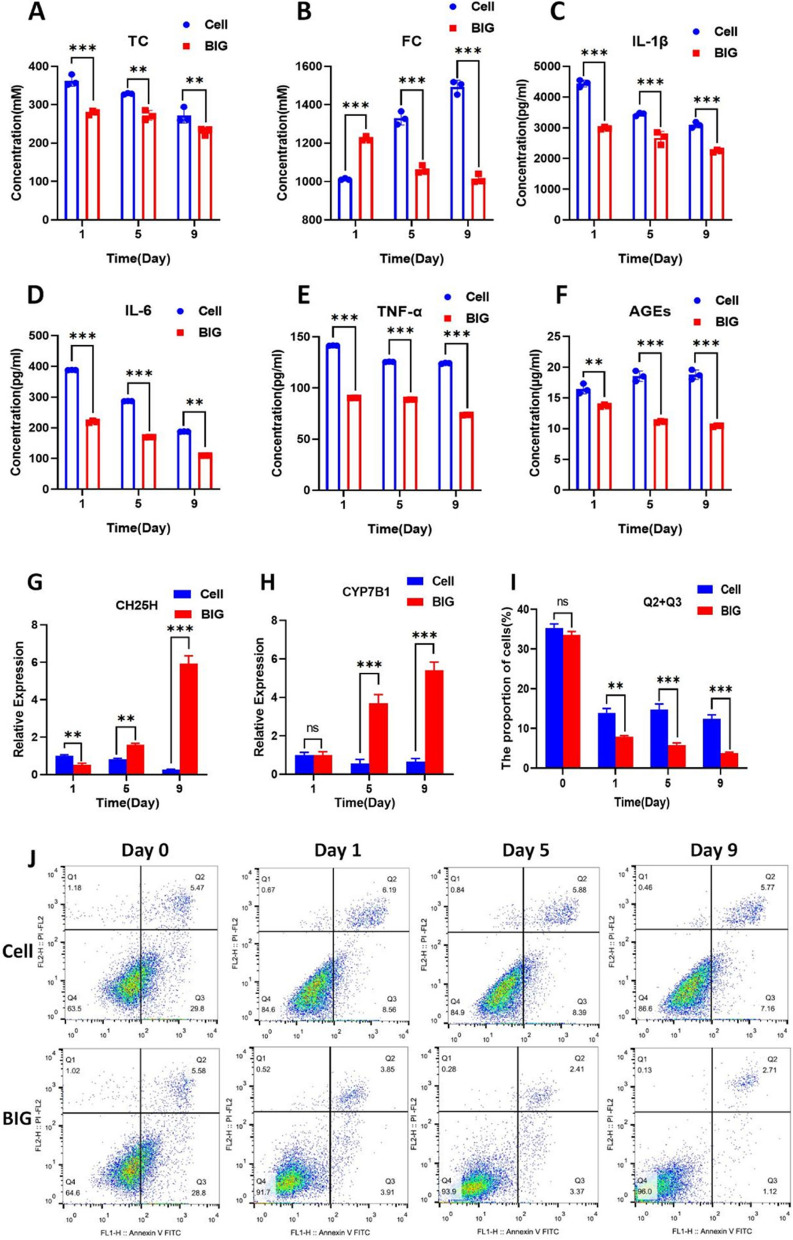
BIG can effectively inhibit the apoptosis of human arthritis chondrocytes by promoting cholesterol metabolism. **A**, **B** BIG promoted cholesterol metabolism, resulting in a decrease in both the total and free cholesterol. **C**–**F** BIG inhibited the expression of IL-1β, IL-6, TNF-α, and AGEs. **G**, **H** BIG stimulated the secretion of the cholesterol metabolism-related enzymes CH25H and CYP7B1. **J** The flow cytometry results show that BIG inhibited the apoptosis of chondrocytes. **I** The proportion of apoptotic cells (Q2 + Q3) was significantly lower in the BIG group than in the control group. Data are presented as the mean ± SEM (*n* = 3) and were analysed by the Kruskal‒Wallis test, **P < 0.01, ***P < 0.001

BIG (400 μg/ml) was cocultured with chondrocytes for 9 days, and the expression levels of IL-1β, IL-6, TNF-α, and advanced glycosylation end products (AGEs) were measured by ELISA. Cholesterol levels were measured biochemically, and CH25H and 25-hydroxycholesterol 7-alpha-hydroxylase (CYP7B1) expression was measured by RT‒qPCR. Our results indicate that BIG promoted cholesterol metabolism by stimulating the secretion of CH25H and CYP7B1, a key rate-limiting enzyme of cholesterol metabolism (Fig. 2G, H), which resulted in a significant reduction in both the total and free cholesterol (Fig. 2A, B). In addition, BIG inhibited the expression of IL-1β, IL-6, TNF-α, and AGEs (Fig. 2C–F). The flow cytometry results show that 400 μg/ml BIG effectively inhibited the apoptosis of chondrocytes (Fig. 2J), and the proportion of apoptotic cells (Q2 + Q3) was significantly lower in the BIG group than in the control group (Cell) on days 1, 5, and 9 (Fig. 2I).

### BIG induces an alteration of total RNA profiles in OA chondrocytes

**Fig. 3:**
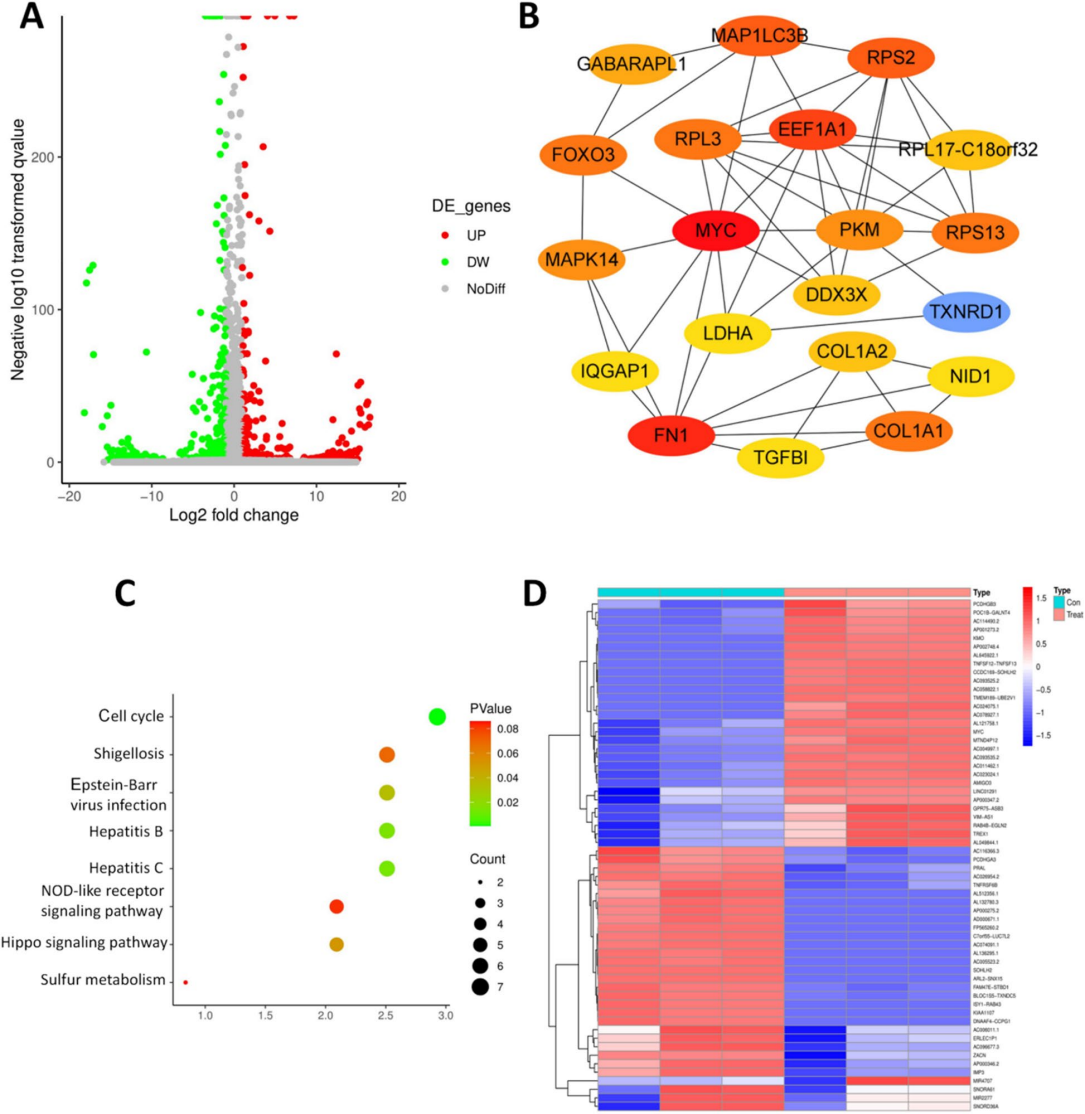
BIG treatment resulted in dysregulated gene levels and interacting signalling in human chondrocytes in OA. **A** Volcano map representing the altered expression levels of the 248 genes caused by BIG treatment. **B** Cytoscape of the top 20 hub genes, and BIG promoted the upregulation of MYC expression. **C** KEGG pathway analysis revealed differentially expressed genes enriched in cell cycle pathways. **D** Differential gene hierarchical clustering analysis between the control and BIG-treated groups was performed, and the results are displayed in a heatmap (*n* = 3, *P* < 0.05). Red indicates upregulated genes, and blue indicates downregulated genes

Human OA chondrocytes were treated with 400 μg/ml BIG for 11 days prior to total RNA sequencing. Among the 27,838 total RNA transcripts analysed, the results showed altered the expression levels of 248 genes, comprising 117 upregulated and 131 downregulated genes (fold change above ± 2, *P* < 0.05) (Fig. 3A). The String database demonstrated the protein‒protein interaction network of the differentially expressed genes, and Cytoscape showed the top 20 hub genes, indicating that BIG promoted increased expression of the MYC gene (Fig. 3B). The KEGG pathway results show differentially expressed genes enriched in cell cycle signalling pathways (Fig. 3C). The heatmap represents the top 60 differentially expressed genes between the control and BIG-treated groups (Fig. 3D).

### BIG causes the upregulation of cell cycle-related proteins and inhibits apoptosis and autophagy to promote the proliferation of chondrocytes in OA

**Fig. 4:**
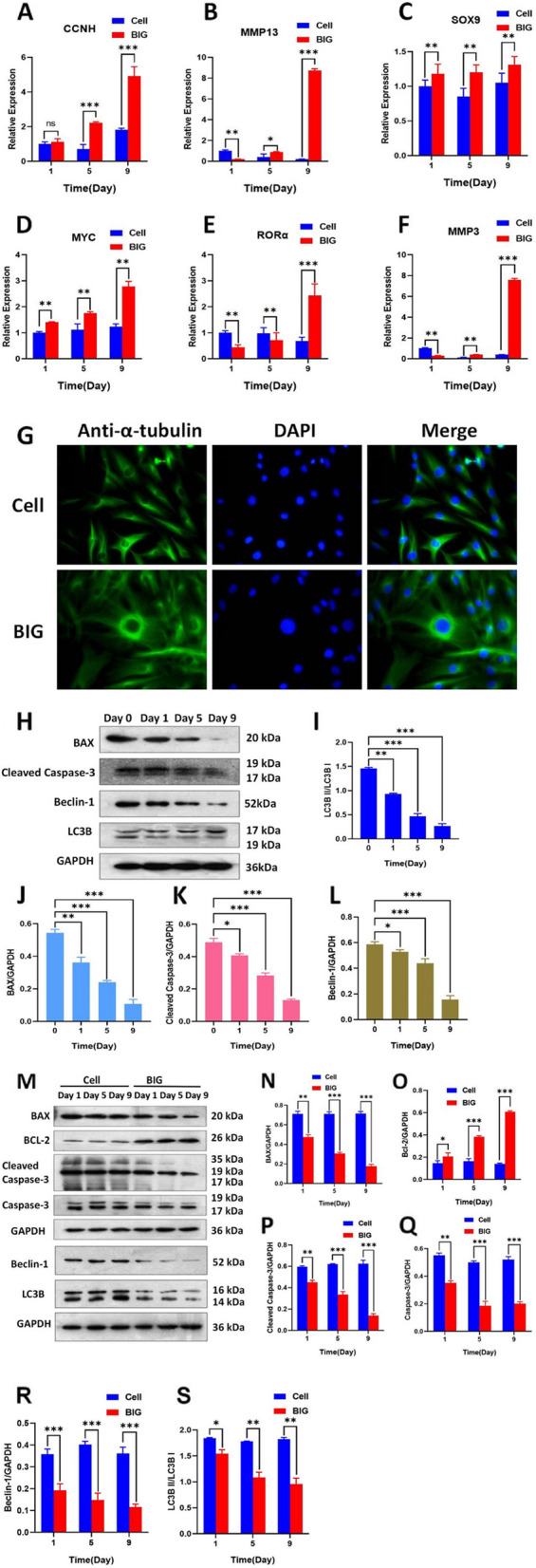
BIG upregulates the expression of cell cycle-related proteins to inhibit apoptosis and autophagy in chondrocytes in OA. **A** BIG promoted the upregulated expression of cyclin CCNH. **B**, **E**, **F** BIG promoted the upregulation of proteins associated with degradation in OA chondrocytes. **C** BIG promoted the upregulation of chondrogenesis-related proteins. **D** BIG effectively caused high expression of the pro-cell division protein MYC. **G** Immunofluorescence confocal images show a peak in cytoskeletal protein α-tubulin replication on day 9 of chondrocyte coculture with BIG (× 400). **H**, **J**–**L** BIG effectively inhibited the expression of BAX, cleaved caspase-3, and Beclin-1. **I** BIG caused a gradual decrease in the autophagy ratio in OA chondrocytes, and the autophagy ratio decreased significantly in the BIG group than in the cell group. **O**–**S** BIG significantly increased Bcl-2 expression but decreased the expression of BAX, cleaved caspase-3, caspase-3, Beclin-1, and LC3B. Data are presented as the mean ± SEM (*n* = 3) and were analysed by the Kruskal‒Wallis test, *P < 0.05, **P < 0.01, ***P < 0.001

We applied 400 μg/ml BIG in coculture with chondrocytes for 9 days to further examine cell cycle-related protein expression by RT‒qPCR. The results show that the expression of cyclin CCNH (Fig. 4A), cell division-promoting protein MYC (Fig. 4D), and articular cartilage degradation-related proteins including RORα, MMP3, MMP13 (Fig. 4E, F, B), and chondrogenic-related protein SOX 9 (Fig. 4C) were significantly increased in the BIG group. Immunofluorescence confocal detection of the cytoskeletal protein α-tubulin and confocal images show that on day 9 of chondrocyte coculture with BIG, a peak in cytoskeletal protein α-tubulin replication was observed in the BIG group compared with the control group (Fig. 4G). We next examined the expression of apoptosis- and autophagy-related proteins. The results show that the expression of the apoptosis-promoting protein BAX, the apoptosis-activated protein cleaved caspase-3, and the autophagy-related protein Beclin-1 decreased in a duration-dependent manner (Fig. 4H). The relative value of LC3B II/LC3B I gradually decreased with the BIG-treated culture time, which confirms that BIG inhibited autophagy in the chondrocytes (Fig. 4I). In addition, we found that BIG increased the expression of the anti-apoptosis-related gene Bcl-2 and gradually decreased the expression of BAX, cleaved caspase-3 (Fig. 4O, A, P) compared with the control group. BIG significantly inhibited autophagy in the human OA chondrocytes (Fig. 4S).

### The c-MYC inhibitor 10058-F4 promotes apoptosis and autophagy in OA chondrocytes

**Fig. 5:**
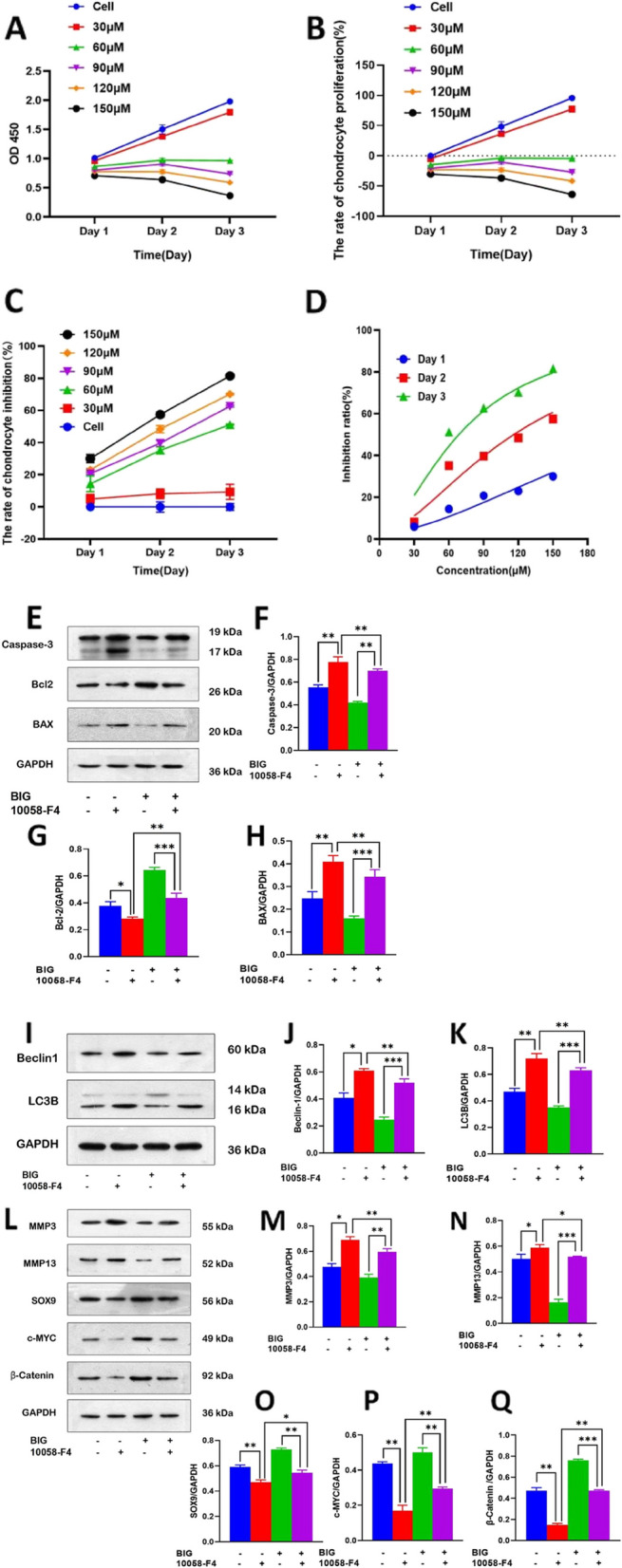
The c-MYC inhibitor 10058-F4 inhibits the proliferation of chondrocytes and promotes apoptosis and autophagy. **A** The c-MYC inhibitor 10058-F4 decreased the concentration of chondrocytes. **B** The proliferation rate of chondrocytes gradually decreased with increasing concentrations of 10058-F4. **C** The inhibition rate of chondrocytes gradually increased with increasing concentrations of 10058-F4. **D** Dose-response curve of 10058-F4 inhibition of chondrocyte proliferation. **E**–**H** 10058-F4 stimulated the proapoptotic protein caspase-3 and increased the expression of BAX, while it inhibited the expression of the antiapoptotic protein Bcl-2. However, BIG can reverse these effects caused by 10058-F4. **I**–**K** 10058-F4 promoted the expression of autophagy-related proteins Beclin-1 and LC3B. **L**–**Q** The c-MYC inhibitor 10058-F4 inhibited the chondrocyte division cycle and that BIG promoted cell division while inhibiting apoptosis. Data are presented as the mean ± SEM (*n* = 3) and were analysed by the Kruskal‒Wallis test. The IC50 of 10058-F4 was analysed by probit regression, *P < 0.05, **P < 0.01, ***P < 0.001

First, we determined the mean inhibitory concentration (IC50) of 10058-F4 in inhibiting chondrocyte proliferation in OA. We treated chondrocytes with five different concentrations of 10058-F4 for 3 days, and the results show that 10058-F4 inhibited chondrocyte proliferation in a duration- and concentration-dependent manner (Fig. 5A). With increasing 10058-F4 concentration, the proliferation rate of chondrocytes decreased significantly (Fig. 5B); however, the inhibition rate of chondrocytes significantly increased (Fig. 5C). Probit regression analysis showed that the IC50 values of 10058-F4 inhibition of chondrocyte proliferation on day 1, day 2, and day 3 were 653.97, 119.38, and 69.63 μM, respectively (Fig. 5D).

We next cocultured chondrocytes with 119.38 μM 10058-F4 for 2 days, detected the expression of apoptosis- and autophagy-related proteins by western blotting and measured the expression of cell cycle-related proteins by PCR. The WB results demonstrate that 10058-F4 promoted the high expression of the apoptotic proteins caspase-3 and BAX; however, the expression of Bcl-2 was decreased (Fig. 5E) and that 10058-F4 also promoted the increased expression of the autophagy-related proteins Beclin-1 and LC3B (Fig. 5I). The WB results also confirm that 10058-F4 promoted the secretion of proteins involved in cartilage degradation, while inhibiting the expression of the chondrogenic-related protein SOX9 as well as the cell division-promoting protein c-MYC (Fig. 5L).

### The c-MYC inhibitor 10058-F4 inhibits the cell cycle via inhibition of cholesterol metabolism

**Fig. 6:**
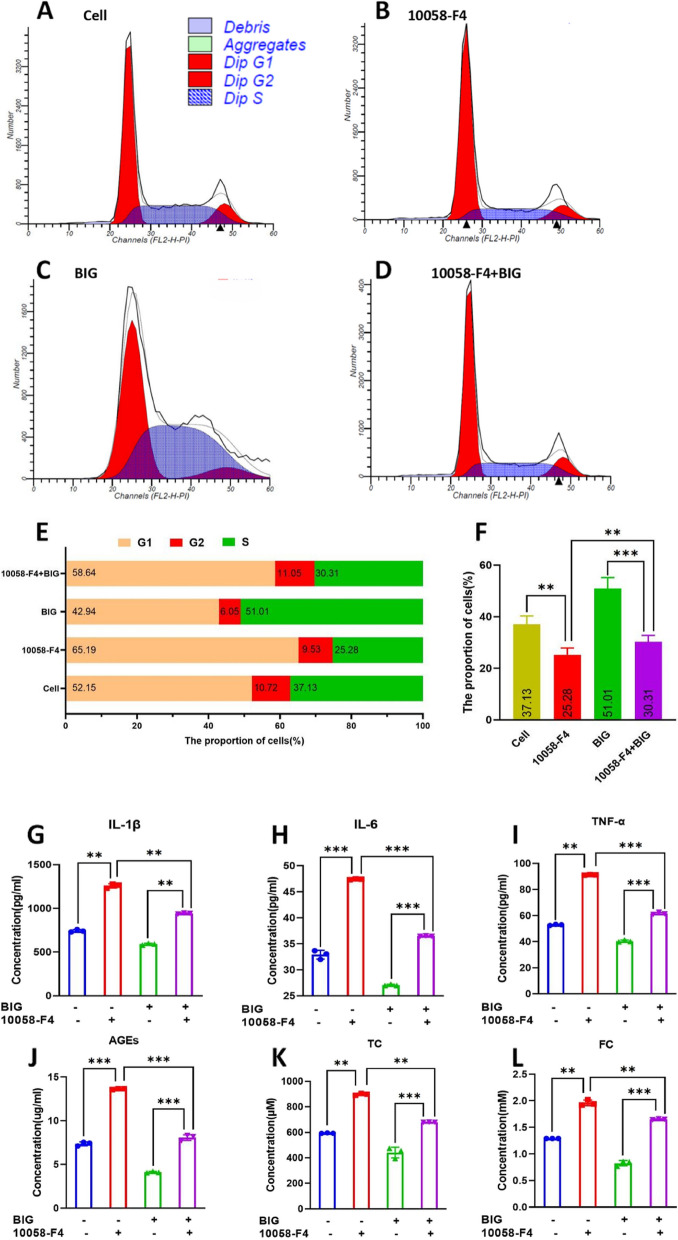
The c-MYC inhibitor 10058-F4 inhibits cholesterol metabolism, which inhibits the cell cycle. Flow cell cycle tests confirmed that 10058-F4 significantly decreased the number of chondrocytes in the S phase. **A** The cell group of chondrocytes was untreated. **B** The c-MYC inhibitor 10058-F4 significantly increased the number of cells in the G1 phase and decreased the number of cells in the S phase. **C** BIG increased the number of chondrocytes in phase S, while the number of cells in phase G1 was significantly decreased. **D** BIG ameliorated the reduced number of cells in phase S caused by 10058-F4, thereby increasing the number of cells in phase S. **E** Bar graph of the proportion of cells in the cell cycle in the four groups. **F** Bar graph of the proportion of cells in phase S. **G**–**J** The c-MYC inhibitor 10058-F4 caused upregulation of IL-1β, IL-6, TNF-α, and AGEs. **K**, **L** 10058-F4 inhibited cholesterol metabolism; however, BIG promoted cholesterol metabolism to reduce the concentrations of TC and FC. Data are presented as the mean ± SEM (*n* = 3) and were analysed by the Kruskal‒Wallis test, **P < 0.01, ***P < 0.001

We divided the chondrocytes into four groups: the cell group of chondrocytes was untreated, the 10058-F4 group of chondrocytes was treated with 119.38 μM 10058-F4 for 2 days, the BIG group of chondrocytes was treated with 400 μg/ml BIG for 2 days, and the 10058-F4+BIG group of chondrocytes was cotreated with 119.38 μM 10058-F4 and 400 μg/ml BIG for 2 days. The results of the flow cell cycle test show that 10058-F4 significantly increased the number of cells in the G1 phase but decreased the number in the S phase (Fig. 6B). BIG significantly increased the number of cells in the S phase (Fig. 6C).

We next determined whether 10058-F4 was correlated with cholesterol metabolism and examined the expression of IL-1β, IL-6, TNF-α, and AGEs and the cholesterol concentration. The ELISA results show that 10058-F4 promoted the increased expression of IL-1β, IL-6, TNF-α, and AGEs (Fig. 6G–J) and inhibited cholesterol metabolism (Fig. 6K, L); in addition, the results confirm that BIG promoted cholesterol metabolism.

### BIG promotes protein-protein interactions

**Fig. 7:**
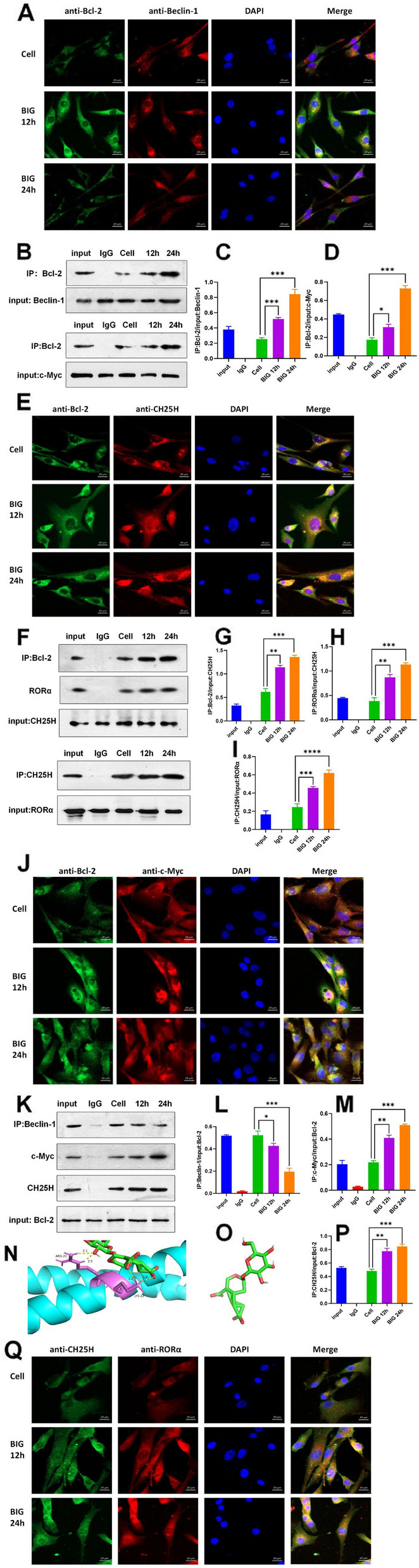
BIG promotes protein-protein interactions. **A**, **E**, **J**, **Q** Sublocalizations of Bcl-2 with Beclin-1, Bcl-2 with CH25H, Bcl-2 with c-MYC, and CH25H with RORɑ were analysed by immunofluorescence assay. Laser confocal microscopy images confirmed that Bcl-2 and Beclin-1, Bcl-2 and CH25H, Bcl-2 and c-MYC, and CH25H with RORɑ were colocalized with the nucleus and in the cell matrix. Chondrocytes pretreated with BIG showed a significantly enhanced fluorescence intensity. The scale bars represent 20 μm. **B**–**D** Chondrocyte lysates were prepared by IP lysis and coimmunoprecipitated with Bcl-2 antibody and IgG as a negative control. The results show the interaction between Bcl-2 with Beclin-1 and c-Myc. **F**–**I** The coimmunoprecipitation results similarly show the CH25H with RORɑ interaction, CH25H with RORɑ and Bcl-2. **K**–**M**, **P** Using c-MYC, Beclin-1, and CH25H as bait proteins for coimmunoprecipitation, the results confirm that Bcl-2 interacted with c-MYC, Beclin-1, and CH25H. O The chemical-molecular structure map of BIG (red: oxygen; green: carbon). **N** Ribbon diagram of the monomeric c-MYC catalytic domain complexed with BIG (pink: the c-MYC active domain). Docking of BIG into the c-MYC catalytic pocket (the map shows the details of molecular docking). Data are presented as the mean ± SEM (*n* = 3) and were analysed by the Kruskal‒Wallis test, *P < 0.05, **P < 0.01, ***P < 0.001

To further explore how BIG prevents apoptosis and autophagy in chondrocytes in OA, we divided chondrocytes into three groups. The cell group was not pretreated, and the other two groups of chondrocytes were pretreated with 400 μg/ml BIG for 12 h or 24 h. The fluorescence intensity of protein interactions was measured by immunofluorescence. The immunofluorescence results show that BIG enhanced the fluorescence of anti-Bcl-2 and anti-Beclin-1, anti-Bcl-2 and anti-CH25H, anti-Bcl-2 and anti-c-Myc, and anti-CH25H and anti-RORɑ (Fig. 7A, E, J, Q). Co-IP demonstrated that Bcl-2 interacts with CH25H, Beclin-1, c-Myc, and RORɑ; moreover, BIG enhanced the interaction between Bcl-2, CH25H, Beclin-1, c-Myc, and RORɑ (Fig. 7B, F, K).

### The crystal structure reveals that BIG binds to the c-MYC catalytic domain

**Fig. 8:**
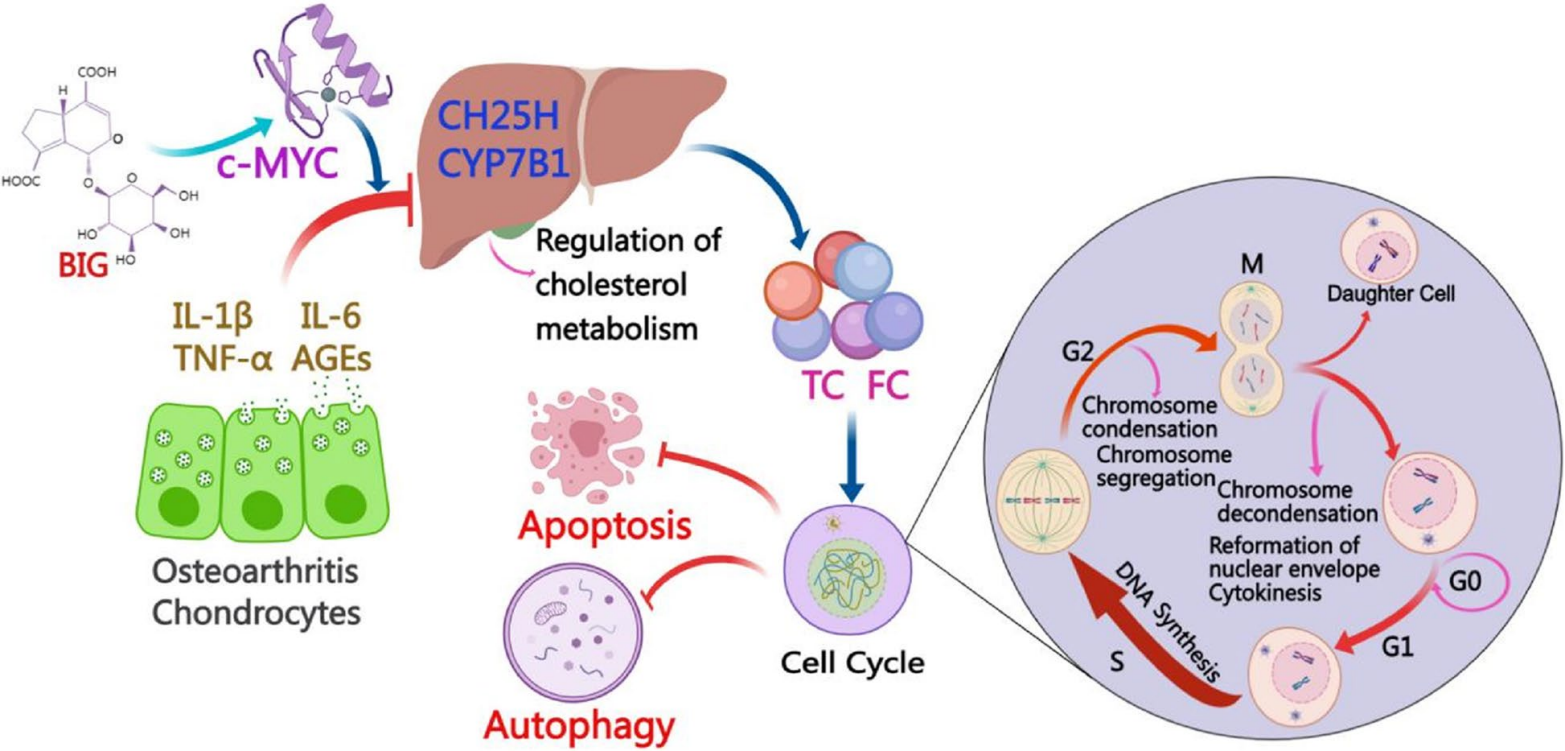
A potential molecular mechanism underlying BIG-induced inhibition of apoptosis and autophagy in chondrocytes in OA. Chondrocytes secrete inflammatory factors, including IL-1β, IL-6, TNF-α, and AGEs, which inhibit the expression of CH25H and CYP7B1 and cause dysregulation of cholesterol metabolism. However, BIG binds to the active domain of c-MYC, and activated c-MYC stimulates CH25H as well as increased CYP7B1 expression, which regulates cholesterol metabolism and promotes the cell cycle of chondrocytes, thus inhibiting chondrocyte apoptosis and autophagy

We derived the crystal structure of the c-MYC catalytic domain complexed with BIG at 1.95-Å resolution. BIG is a single compound extracted from the borojoa fruit, and its molecular structure is shown in Fig. 7O. Its chemical formula is C_16_H_20_O_11_, and its exact mass is 388.10. The autodock results show that BIG fits well into the c-MYC catalytic domain and has a binding energy of − 5.65 kcal/mol. The O-1 of BIG forms two hydrogen bonds of 2.1 and 2.5 Å with the amide nitrogen of the ARG-21 side chain, the O-9 of BIG forms a hydrogen bond of 2.2 Å with the hydroxy of the LYS-22 sidechain, and O-10 of BIG forms a hydrogen bond of 1.8 Å with the amide nitrogen of the LYS-22 side chain (Figs. 7N and 8).

## Discussion

The present study showed that the main active component from borojoa, BIG, inhibited chondrocyte apoptosis and autophagy in patients with OA, and this effect was mediated by an effect on BIG-promoted cholesterol metabolism. To our knowledge, this is the first study of BIG in protecting against OA. Our results have important implications for the development of iridoids from natural plants as potential and safe therapeutic agents and their use in degenerative diseases such as OA. Although drugs that regulate OA fate and promote chondrocyte generation have been extensively developed, their clinical applications remain a long way from realization. To date, few reports have been published on BIG from borojoa and its extracts in the treatment of OA. The natural iridoid BIG has the advantages of regulating OA fate and promoting chondrocyte proliferation, helping to restore the normal function of chondrocytes. It is derived from the fruit of a natural plant and can be safely consumed by humans. Thus, patients with OA could intake natural iridoids to promote chondrocyte proliferation and improve OA function with minimal side effects. Moreover, it is critical to understand the mechanism of action of BIG treatment in OA, which may contribute to the understanding of chondrocyte biology and acceleration of new drug development.

OA is considered a degenerative disease and affects 18% of females and 10% of males over the age of 60 globally [23, 24]. Currently, therapies for OA are insufficient and unsatisfactory, and there is an urgent need for the development of DMOADs [25]; however, there are no convincing DMOADs available for OA [26–28].

In this study, we extracted the small molecule BIG from *Borojoa patinoi* Cuatrec, and the results confirm that BIG enhanced the proliferation of chondrocytes and decreased cell apoptosis and autophagy, which increased our interest in further investigation to explore the specific mechanism by which BIG promotes chondrocyte proliferation. Our results show that BIG suppressed the expression of IL-1β, IL-6, TNF-α, and AGEs while stimulating the secretion of CH25H and CYP7B1, which resulted in increased expression of FC and TC. The flow cytometry results show that BIG effectively inhibited the apoptosis of chondrocytes. Furthermore, BIG inhibited the expression of the autophagy-related protein Beclin-1. Some previous studies have elaborated that cholesterol plays an important role in physiological processes, including development, homeostasis, ageing, and even cell death [29, 30]. Cholesterol metabolism is closely associated with the pathogenesis of OA [31–33]. Some reports have shown that the CH25H–CYP7B1–RORα axis of cholesterol metabolism can regulate OA [34]. Overexpression of CH25H or CYP7B1 in mouse joint tissues resulted in experimental cartilage degradation. The levels of cholesterol in OA chondrocytes were increased. Despite the potential effects of statins in treating OA [35, 36], they have also been shown to have toxic side effects, such as decreased liver function and muscle pain. Here, our results show that BIG has strong antioxidant capacity, can significantly reduce total cholesterol and free cholesterol in OA chondrocytes, and can upregulate the expression of CH25H and CYP7B1. Therefore, we hypothesized that BIG regulates the phenotype of chondrocytes partly through cholesterol metabolism.

To search for key proteins that BIG acts on in chondrocytes in OA, we treated chondrocytes with 400 μg/ml BIG and extracted total RNA for sequencing. Our results show that BIG inhibited chondrocyte apoptosis by upregulating the expression of MYC, and KEGG analysis revealed differentially expressed genes enriched in cell cycle signalling pathways; there were five upregulated genes enriched in the cell cycle signalling pathway, including YWHAB (Log_2_ FC = 1.53), YWHAH (Log_2_ FC = 1.28), CCNH (Log_2_ FC = 1.22), HDAC2 (Log_2_ FC = 1.20), and CDK2 (Log_2_ FC = 1.47). The results of RT-qPCR confirmed that BIG upregulated CCNH expression (Fig. 4A), and BIG inhibited the expression of pro-inflammatory cytokines, such as IL-1β, IL-6, and TNF-α (Fig. 2C–E). However, BIG affects the expression of MMPs in a time-dependent manner, and BIG downregulates the expression of MMPs on the first day, and from the fifth day later, BIG promotes the expression of MMPs (Fig. 4B, F). Flow cell cycle testing confirmed that BIG prompted a significant increase in the number of chondrocytes in the S phase, and BIG accelerated cell proliferation while promoting DNA synthesis in chondrocytes. The c-MYC inhibitor 10058-F4 inhibits the proliferation of chondrocytes by promoting apoptosis and autophagy. 10058-F4 significantly increased the expression of IL-1β, IL-6, TNF-α, and AGEs, while the increased concentrations of TC and FC due to cholesterol metabolism were suppressed, which upregulated the expression of apoptosis- and autophagy-related proteins caspase-3, BAX, Beclin-1, and LC3B and downregulated the expression of the anti-apoptosis-related protein Bcl-2. Bcl-2 is a primary anti-apoptotic factor in its family and is expressed in various cell types [37–39]. Previous studies in mice and humans revealed the roles of Bcl-2 in apoptosis [40–42]. Increased expression of Bcl-2 could decrease cell apoptosis. Bcl-2 overexpression has been associated with inhibition of BAX-mediated apoptosis [43, 44]. In this study, we revealed that BIG increased Bcl-2 expression and decreased BAX expression to regulate chondrocyte apoptosis. In addition, our results also confirm that 10058-F4 inhibited the expression of the cell division-related protein c-MYC, and the number of cells in the S phase of the cell cycle was significantly reduced, which inhibited cell proliferation.

In the current results, we also found that BIG could regulate cholesterol metabolism; however, whether Bcl-2 or c-MYC is correlated with cholesterol metabolism remains unknown. Moreover, few studies have reported the potential link between apoptosis and cholesterol metabolism in OA. Therefore, a Co-IP assay was employed to test the physical interactions between Bcl-2 with CH25H, Bcl-2 with Beclin-1, and Bcl-2 with c-MYC proteins. The Co-IP assay showed that Bcl-2 interacted with CH25H, while c-MYC interacted with Bcl-2 at 12 and 24 h after BIG treatment. BIG enhanced the Bcl-2 and c-MYC interaction but also enhanced the Bcl-2 and CH25H interaction compared to the untreated cells. The molecular docking results demonstrate well docking of BIG into the catalytic domain of c-MYC and a binding energy of − 5.65 kcal/mol. These findings identify the underlying mechanism by which BIG enhances the c-MYC-mediated regulation of CH25H on cholesterol metabolism to inhibit chondrocyte apoptosis in vitro, suggesting that BIG may be a promising new drug worth developing for patients with OA. BIG may be a potential strategy for treating cholesterol metabolism-related diseases such as OA similar to statins but without complications, including the muscle pain associated with statins, but more investigation is needed.

The main limitation of this study is no direct evidence that BIG promotes cholesterol metabolism, for example, adenoviral overexpression of cholesterol hydroxylase CH25H or CYP7B1, the effect of BIG on cholesterol metabolism. In addition, knockout or knockdown of CH25H or CYP7B1, whether BIG improves OA. However, we used the c-MYC inhibitor 10058-F4 to inhibit cholesterol metabolism, whereas BIG can reverse this effect, and this result at least suggests that BIG improving OA is associated with cholesterol metabolism.

## Conclusions

In conclusion, we showed that the active component BIG from borojoa promotes chondrocyte proliferation and reduces apoptosis and enhances extracellular matrix synthesis in vitro, which is associated with cholesterol metabolism. BIG regulates cholesterol metabolism in chondrocytes by promoting the interaction of c-MYC and Bcl-2 as well as Bcl-2 and CH25H.

These findings suggest that BIG might be a potential new drug to regulate cholesterol metabolism and treat disorders of cholesterol metabolism, such as OA, without toxic side effects.

### Supplementary Information

**Additional file 1.**

## Data Availability

The datasets used and/or analyzed in the current study are available from the corresponding author upon reasonable request.

## Abbreviations

OA: Osteoarthritis
BIG: Borojoa iridoid glycoside
YLD: Years lived with disability
DMOADs: Disease-modifying osteoarthritis drugs
OD: Optical density
TC: Total cholesterol
FC: Free cholesterol
AGEs: Advanced glycosylation end products
COL2A-1: Collagen, type II, alpha-1
FITC/PI: Annexin V-fluorescein isothiocyanate/propidium iodide
CCK8: Cell Counting Kit-8
RT‒qPCR: Real-time quantitative PCR
CH25H: Cholesterol-25-hydroxylase
CYP7B1: 25-Hydroxy-cholesterol 7-alpha-hydroxylase
MMPs: Matrix metalloproteinases
