# Supplementary figures and images for: 4,8-Dicarboxyl-8,9-iridoid-1-glycoside inhibits apoptosis in human osteoarthritis chondrocytes via enhanced c-MYC-mediated cholesterol metabolism in vitro
Source: Arthritis Res Ther. 2023 Dec 11;25:240. doi: 10.1186/s13075-023-03217-1 (PMC10712063; doi:10.1186/s13075-023-03217-1)

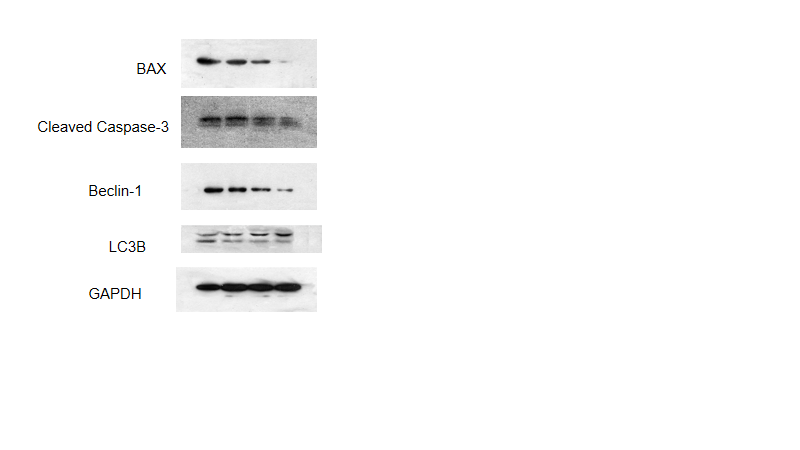


**Figure 4.H**


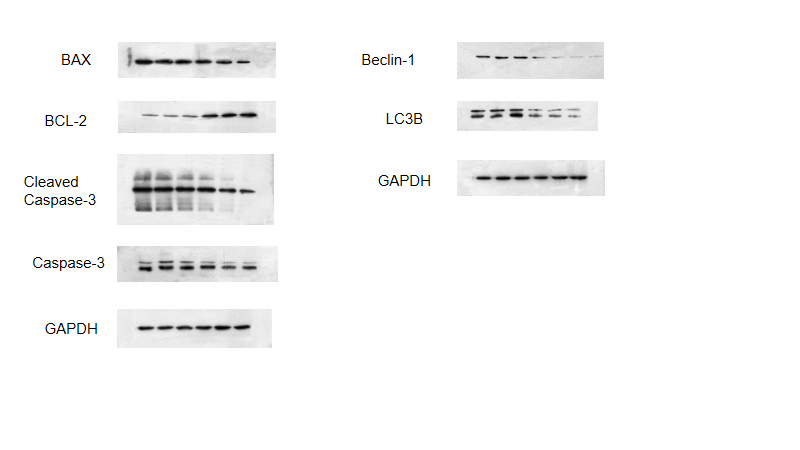


**Figure 4M.**


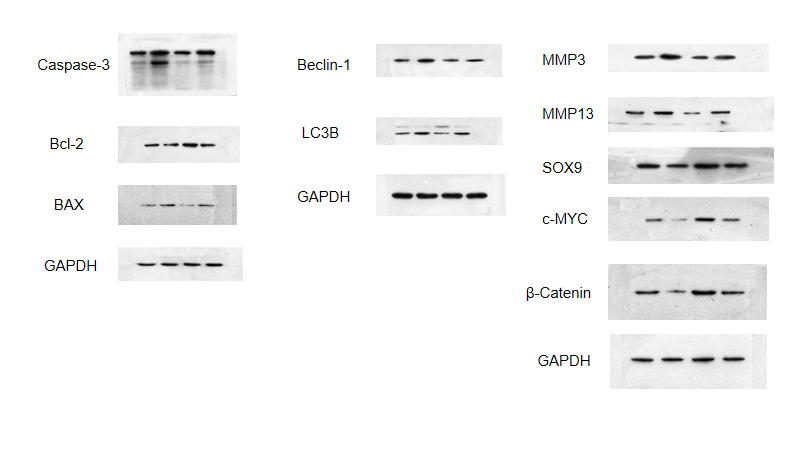


**Figure 5.E.**


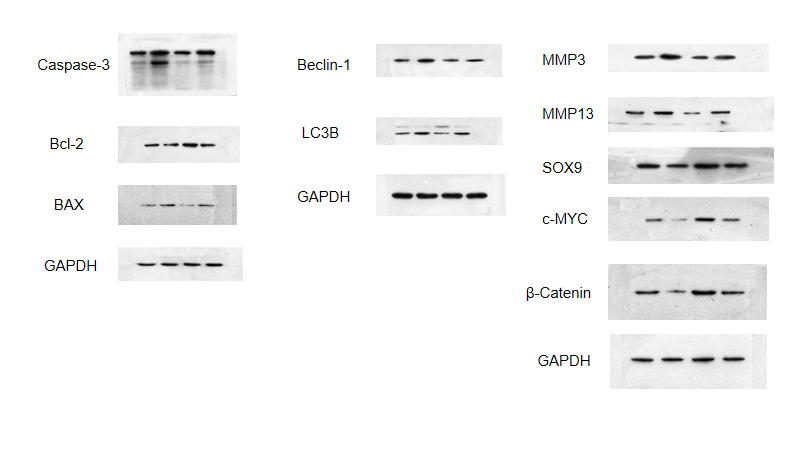


**Figure.5.I**


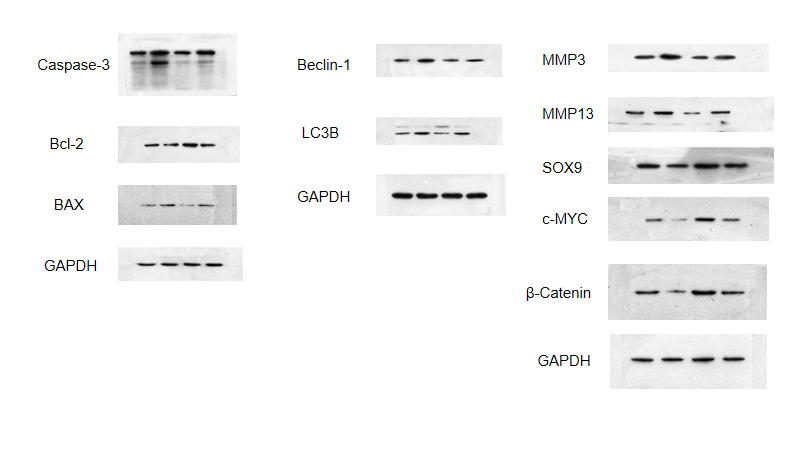


**Figure 5.L**


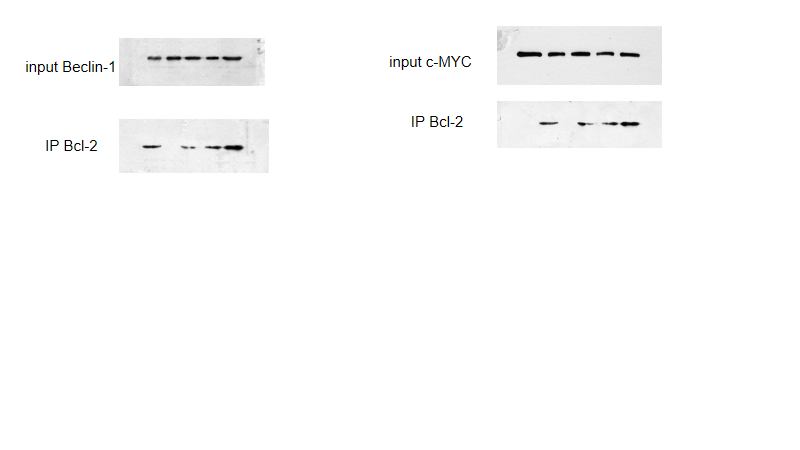


**Figure 7.B**


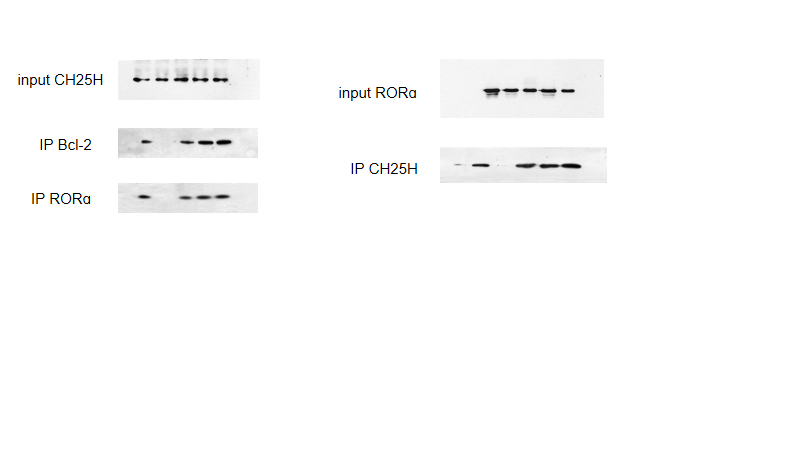


**Figure 7.F**


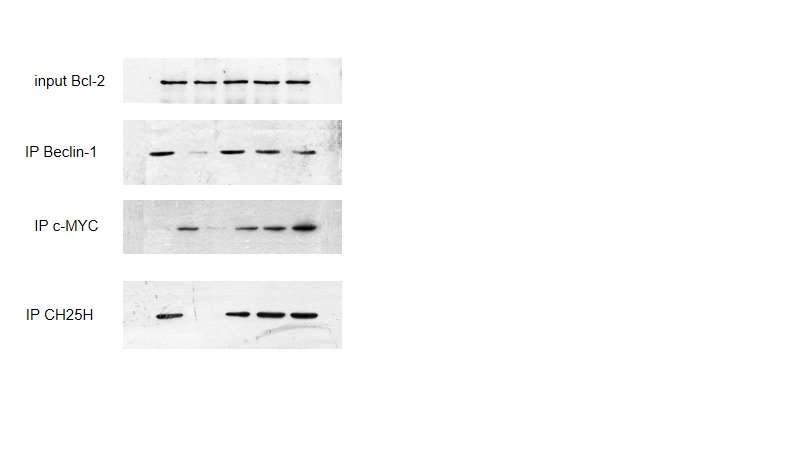


**Figure 7.K**

Supplement: Supplementary file 1 — Additional file 1. [file 13075_2023_3217_MOESM1_ESM.docx]
